# Migration of non-absorbable polymer clips in hepato-biliary-pancreatic surgery: a report of four cases

**DOI:** 10.1186/s40792-021-01269-6

**Published:** 2021-08-14

**Authors:** Yukari Kihara, Yutaka Takeda, Yoshiaki Ohmura, Yoshiteru Katsura, Go Shinke, Ryo Ikeshima, Shinsuke Katsuyama, Kenji Kawai, Masayuki Hiraki, Keijiro Sugimura, Toru Masuzawa, Atsushi Takeno, Taishi Hata, Kohei Murata

**Affiliations:** Department of Surgery, Kansai Rosai Hospital, 3-1-69, Inabaso, Amagasaki, Hyogo 660-8511 Japan

**Keywords:** Hem-o-Lok, Non-absorbable polymer clip, Migration, Laparoscopic surgery

## Abstract

**Background:**

Ligation clips are used for vessel or tissue ligation in surgery. Although previous reports have described the migration of metallic clips after hepato-biliary-pancreatic surgery, very few reports have described the migration of non-absorbable polymer clips (NAPCs: Hem-o-Lok).

**Case presentation:**

We present 4 cases of NAPC migration that occurred after laparoscopic surgery. Case 1 was an 81-year-old woman that had undergone a laparoscopic right hemihepatectomy for an intrahepatic bile duct cyst adenocarcinoma at the age of 79 years. Two years after the operation, she underwent an upper gastrointestinal endoscopy to investigate epigastric pain. The endoscopy showed NAPCs lodged at the anterior side of the duodenal bulb. Case 2 was an 80-year-old man that had undergone a laparoscopic cholecystectomy for choledocholithiasis at the age of 77 years. Three years after the operation, follow-up computed tomography and magnetic resonance cholangiopancreatography (MRCP) imaging indicated a mass in the upper bile duct. After a laparoscopic bile duct resection and reconstruction, an NAPC was found inside the inflammatory pseudotumor. Case 3 was a 63-year-old man that had undergone laparoscopic liver S4b and S5 resections and lymph node dissection for gallbladder cancer. Three months after the operation, follow-up MRCP imaging suggested a bile duct stenosis. An endoscopic retrograde cholangiopancreatography (ERCP) was performed, and an NAPC was found inside the bile duct. Case 4 was a 74-year-old man that had undergone a laparoscopic S5 segmentectomy, S7 partial liver resection, and cholecystectomy for liver metastasis of lung cancer and cholelithiasis. A trans-cystic drainage tube was inserted, and it was ligated and fixed with NAPCs. Three months after the operation, follow-up MRCP imaging showed common bile duct stones (CBDS). An ERCP was performed, and two NAPCs were found with the CBDS.

**Conclusions:**

Few previous reports have described complications due to NAPC migration after hepato-biliary-pancreatic surgery. However, with the widespread use of NAPC, postoperative complications due to NAPC migration are expected to increase in the near future. The differential diagnosis of complications should include potential NAPC migration in patients that have undergone laparoscopic surgery.

## Background

Surgical resections often require the use of metal or polymer clips to ligate tissues and blood vessels. The widespread adoption of laparoscopic hepato-biliary-pancreatic surgery has led to an increase in the use of non-absorbable polymer clips (NAPCs: Hem-o-Lok). NAPCs provide a secure ligation, and they are both non-conducive and radiolucent. Although a number of previous reports have described metal clip migration in the hepato-biliary-pancreatic field, very few reports have described NAPC migration. Herein, we describe four cases of NAPC migration.

This study was approved by the Human Ethics Review Committee of Kansai Rosai Hospital (Certificate Number: 2102001). It was conducted in accordance with the Declaration of Helsinki. Each patient provided signed informed consent.

## Case presentation

### Case 1

**Fig. 1:**
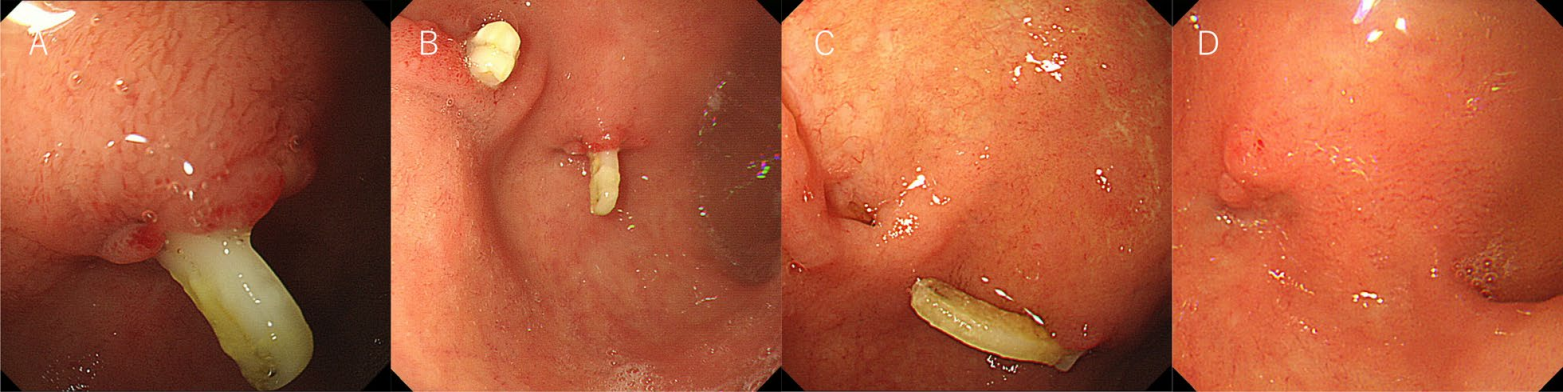
Upper gastrointestinal endoscopy after a laparoscopic right hemihepatectomy. **A** Upper gastrointestinal (GI) endoscopy shows 1 medium-large (ML) NAPC at the anterior aspect of the duodenal bulb; 2 years after the operation. **B** Follow up GI endoscopy performed 2 months after the first upper GI endoscopy shows 2 ML NAPCs at the anterior aspect of the duodenal bulb. **C** Follow up GI endoscopy performed 5 months after the first upper GI endoscopy shows 1 ML NAPC at the anterior aspect of the duodenal bulb. **D** The NAPC fell out, without any procedure, after 7 months of observation

An 81-year-old woman had previously undergone a laparoscopic right hemihepatectomy for intrahepatic bile duct cyst adenocarcinoma at the age of 79 years. NAPCs were used in the operation. She was followed up in the post-operative outpatient clinic. Two years after the operation, she underwent an upper gastrointestinal (GI) endoscopy to investigate a complaint of epigastric pain. The results showed one medium–large (ML, 1.0 cm) NAPC at the anterior side of the duodenal bulb (Fig. 1A). The patient was given a proton pump inhibitor, which relieved the epigastric pain. Follow-up GI tests were performed 2 months after the first upper GI endoscopy. These tests revealed two ML NAPCs at the duodenal bulb (Fig. 1B).

**Fig. 2:**
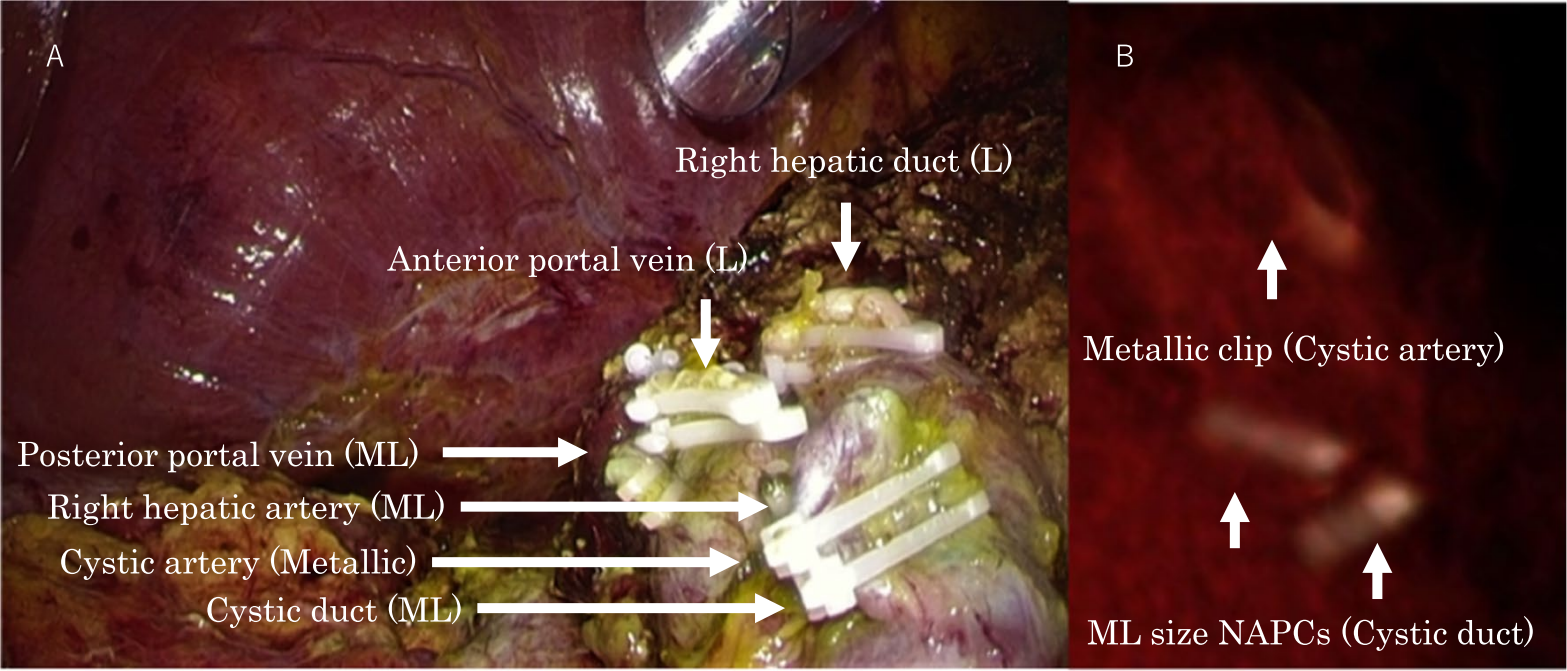
Intraoperative findings of a laparoscopic right hemihepatectomy and CT imaging of the duodenal bulb. **A** A laparoscopic right hemihepatectomy had been performed for an intrahepatic bile duct cystadenocarcinoma. Medium-large (ML) NAPCs were placed at the cystic duct, right hepatic artery, and the posterior branch of the portal vein. Large NAPCs were placed at the right hepatic duct and at the anterior and posterior branches of the portal vein. A metal clip was placed at the cystic artery. **B** Reconstructed CT imaging showed two ML NAPCs on the duodenal bulb and one metal clip nearby

In the previous operation, ML NAPCs had been placed at the cystic duct, the right hepatic artery, and the posterior branch of the portal vein. In addition, large (L, 1.3 cm) NAPCs were placed at the right hepatic duct and the anterior and posterior branches of the portal vein. A metal clip was placed at the cystic artery (Fig. 2A).

When the migrated NAPC was found in the duodenal bulb, a computed tomography (CT) scan was performed. Reconstruction CT imaging showed two ML NAPCs at the duodenal bulb and one metallic clip nearby (Fig. 2B). Considering the distance that the metal clip migrated from its placement on the gallbladder artery, we concluded that the ML NAPCs used to clip the cystic duct must have migrated to the duodenal wall. The patient characteristics and results are summarized in Table 1.

**Table 1:** Patient Characteristics and Results

Case	Age	Gender	Diagnosis	Operation	Duration of Migration after Initial Surgery	Migrated site	Symptoms	Treatment for the migrated clips
1	81	Female	Bile duct cystadenocarcinoma	Laparoscopic right hemihepatectomy	2 years	Duodenal bulb	Epigastric pain	Disappeared Spontaneously
2	80	Male	Cholecystolithiasis	Laparoscopic cholecystectomy	3 years	Comon bile duct	Asymptomatic (Inflammatory pseudotumor)	Laparoscopic bile duct resectionand reconstruction
3	63	Male	Gallbladder cancer	Laparoscopic liver S4b and S5 resection and lymph node dissection	3 months	Comon bile duct	Asymptomatic (Bile duct stenosis)	Removed by ERCP
4	74	Male	Liver metastasis from lung cancer	Laparoscopic S5 and S7 liver resection and cholecystectomy	3 months	Comon bile duct	Asymptomatic (Bile duct stone)	Removed by ERCP

### Case 2

**Fig. 3:**
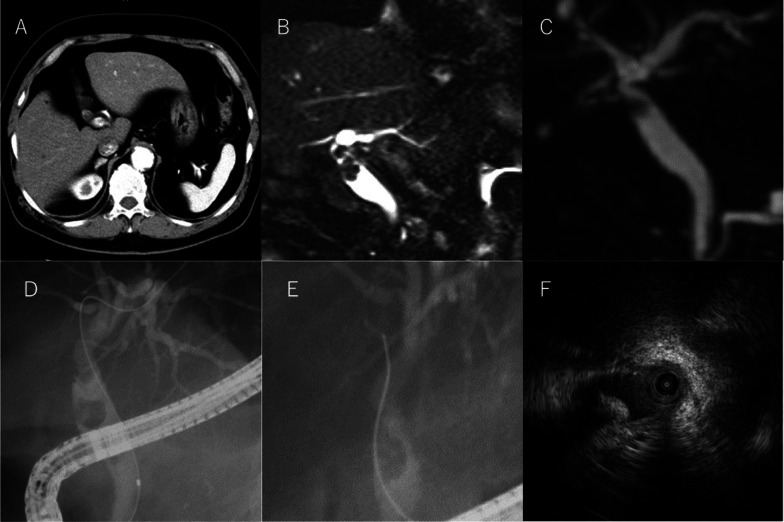
Preoperative findings of choledocholithiasis in "Case 2". **A** CT image shows a mass located in the upper bile duct. **B**, **C** MRCP imaging shows a mass located in the upper bile duct. **D**, **E** ERCP image shows a filling defect in the upper bile duct, which suggests bile duct cancer. **F** Intraductal ultrasonography image reveals an intraductal tumor

**Fig. 4:**
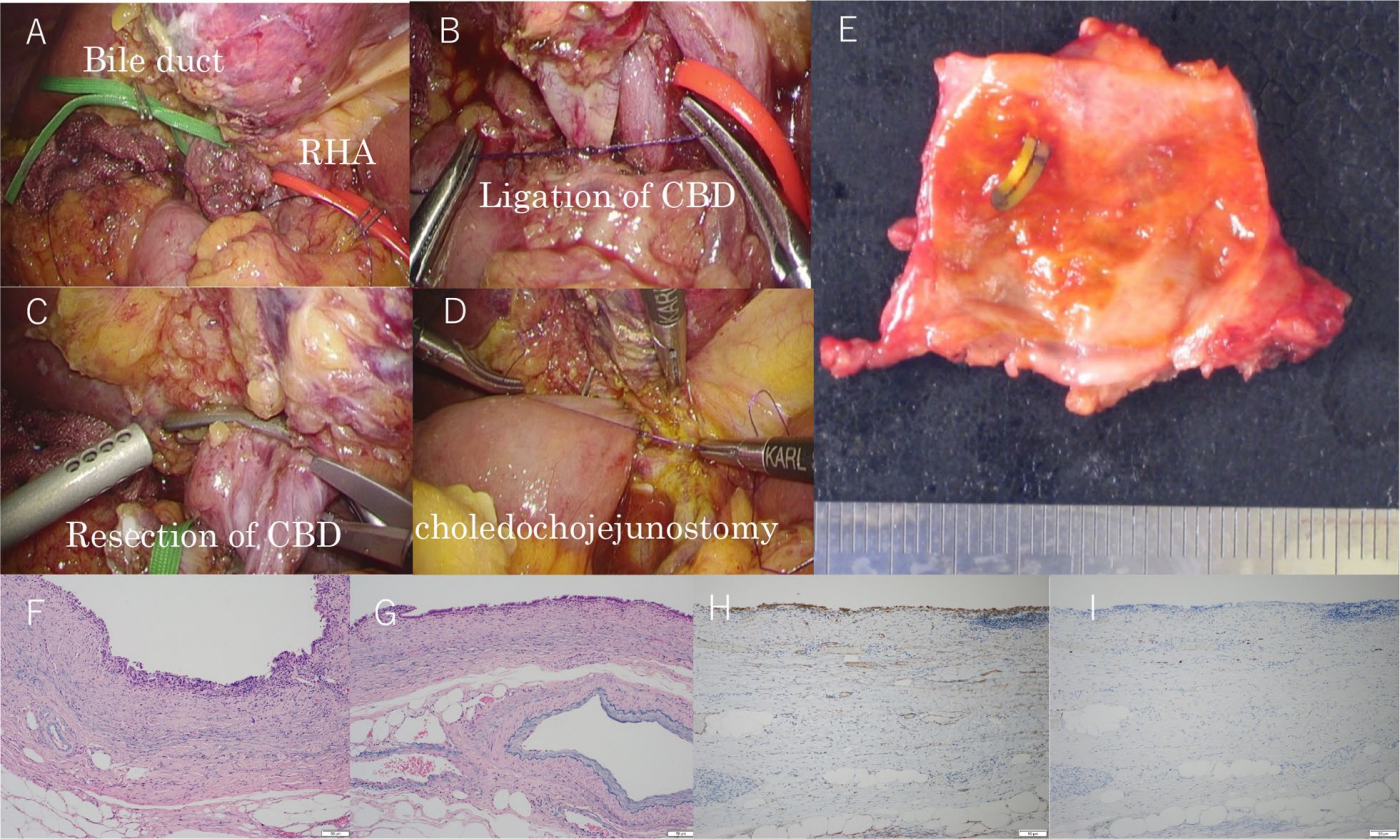
Intraoperative and postoperative findings for case 2. **A**–**D** Images show a laparoscopic bile duct resection and reconstruction. **E** An NAPC is shown inside the tumor. **F**, **G** Victoria blue–hematoxylin and eosin staining indicates erosion of the bile duct, fibroblast proliferation, and inflammatory cell infiltration. **H** α-SMA staining shows positive expression. **I** Desmin staining was positive expression

An 80-year-old man had previously undergone a laparoscopic cholecystectomy and endoscopic choledocholithotomy for choledocholithiasis at the age of 77 years. NAPCs had been placed at the cystic artery and cystic duct. Three years after the operation, follow-up imaging with CT and magnetic resonance cholangiopancreatography (MRCP) indicated a mass in the upper bile duct (Fig. 3A–C), which was suspected to be bile duct cancer. The patient was referred to our hospital for surgical treatment. We performed endoscopic retrograde cholangiopancreatography (ERCP), which also indicated a mass located in the upper bile duct (Fig. 3D, E). Intraductal ultrasonography revealed an intraductal tumor (Fig. 3F). A laparoscopic bile duct resection and reconstruction were performed (Fig. 4A–D), and one NAPC was found inside the tumor (Fig. 4E). In a histochemical analysis of the tumor, Victoria blue–hematoxylin and eosin (VB-HE) staining showed excessive myofibroblast and fibroblast proliferation, with chronic inflammatory cell infiltration (Fig. 4F, G). Immunohistochemical stains showed positive α-smooth muscle actin and desmin expression (Fig. 4H, I). The pathological diagnosis was an inflammatory pseudotumor (IPT), caused by the migrated NAPC.

### Case 3

**Fig. 5:**
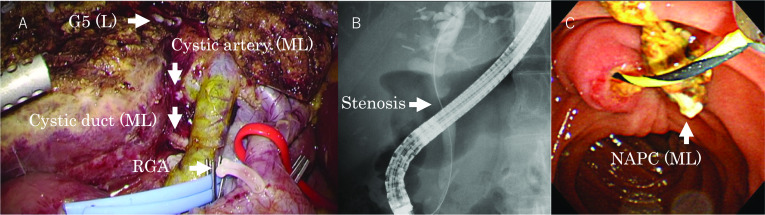
Intraoperative and postoperative findings for case 3. **A** Laparoscopic S4b/S5 resection and lymph node dissection were performed. Medium-large NAPCs were placed at the cut surface of the liver, the cystic artery, the cystic duct, and the right gastric artery. Large NAPCs were placed at the Glissonean pedicle of segment 5. **B** ERCP and endoscopic biliary drainage were performed. **C** A medium-large NAPC was found when the endoscopic biliary drainage tube was removed

A 63-year-old man had undergone a laparoscopic right hemicolectomy for appendix cancer and a cholecystectomy for adenomyomatosis of the gallbladder. The pathological diagnosis was gallbladder cancer, and he was referred to our hospital for a liver resection. He underwent laparoscopic liver S4b and S5 resections and lymph node dissections. ML NAPCs were placed at the cut surface of the liver, the cystic artery, the cystic duct, and the right gastric artery (Fig. 5A). In addition, L NAPCs were placed at the Glissonean pedicle of segment 5. Three months after the operation, a follow-up MRCP suggested that bile duct stenosis had formed, and another ERCP was performed (Fig. 5B). A NAPC was found when the endoscopic biliary drainage tube was removed (Fig. 5C). Based on the endoscopic images, the migrated NAPC was about 1 cm in size; thus, we presumed it was an ML NAPC. We concluded that the NAPCs used to clip the cystic duct had migrated into the common bile duct. After the NAPC was removed, balloon dilatations were performed twice, and the stenosis disappeared. Currently, the patient is under observation without stenting.

### Case 4

**Fig. 6:**
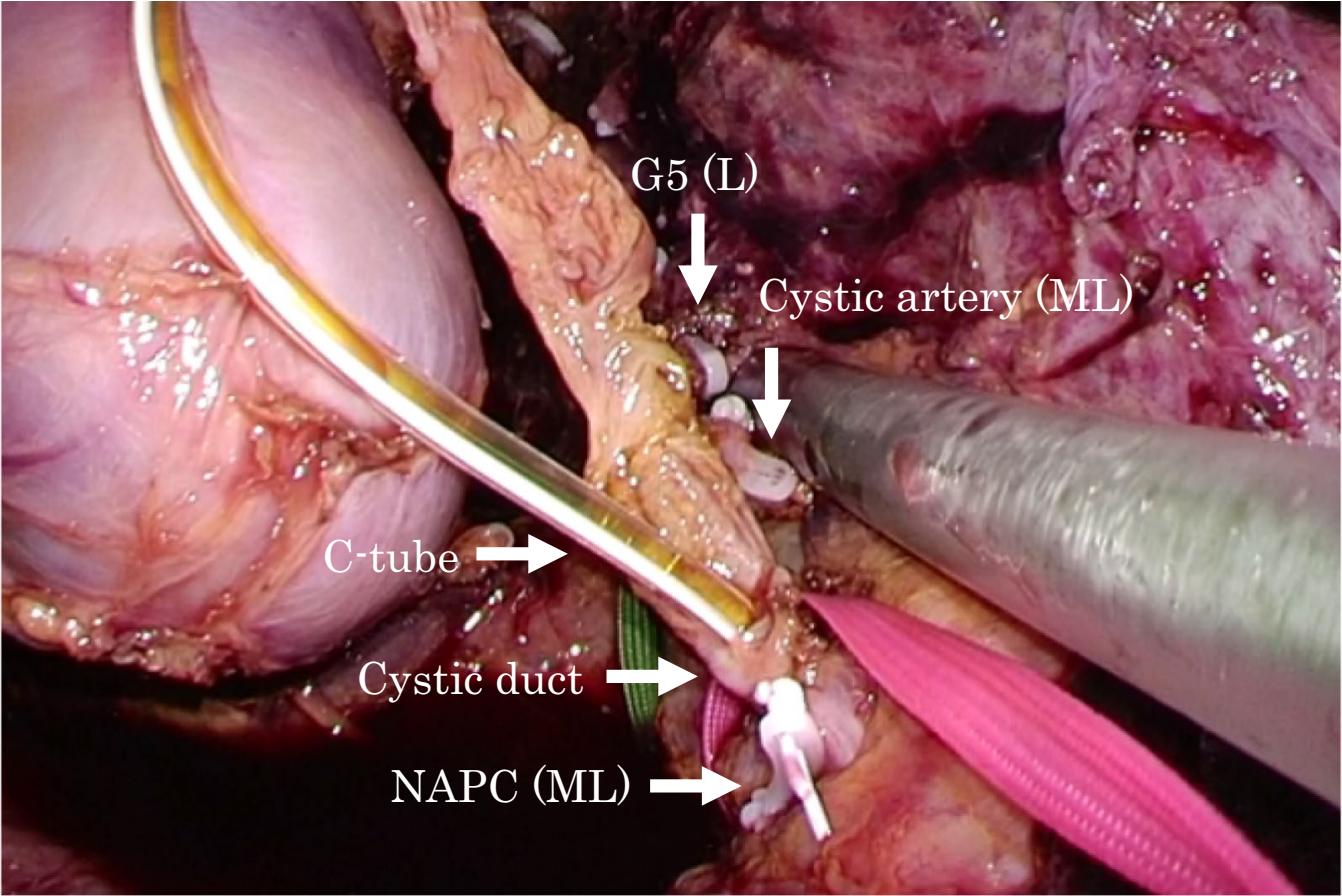
Intraoperative findings for case 4. A laparoscopic S5 segmentectomy, S7 partial liver resection, and cholecystectomy were performed for liver metastasis from lung cancer and cholelithiasis. Medium-large NAPCs were placed at the cut surface of the liver and the cystic artery. Large NAPCs were placed at the Glissonean pedicle of segment 5. A trans-cystic drainage tube was inserted, and it was ligated and fixed with medium-large NAPCs

**Fig. 7:**
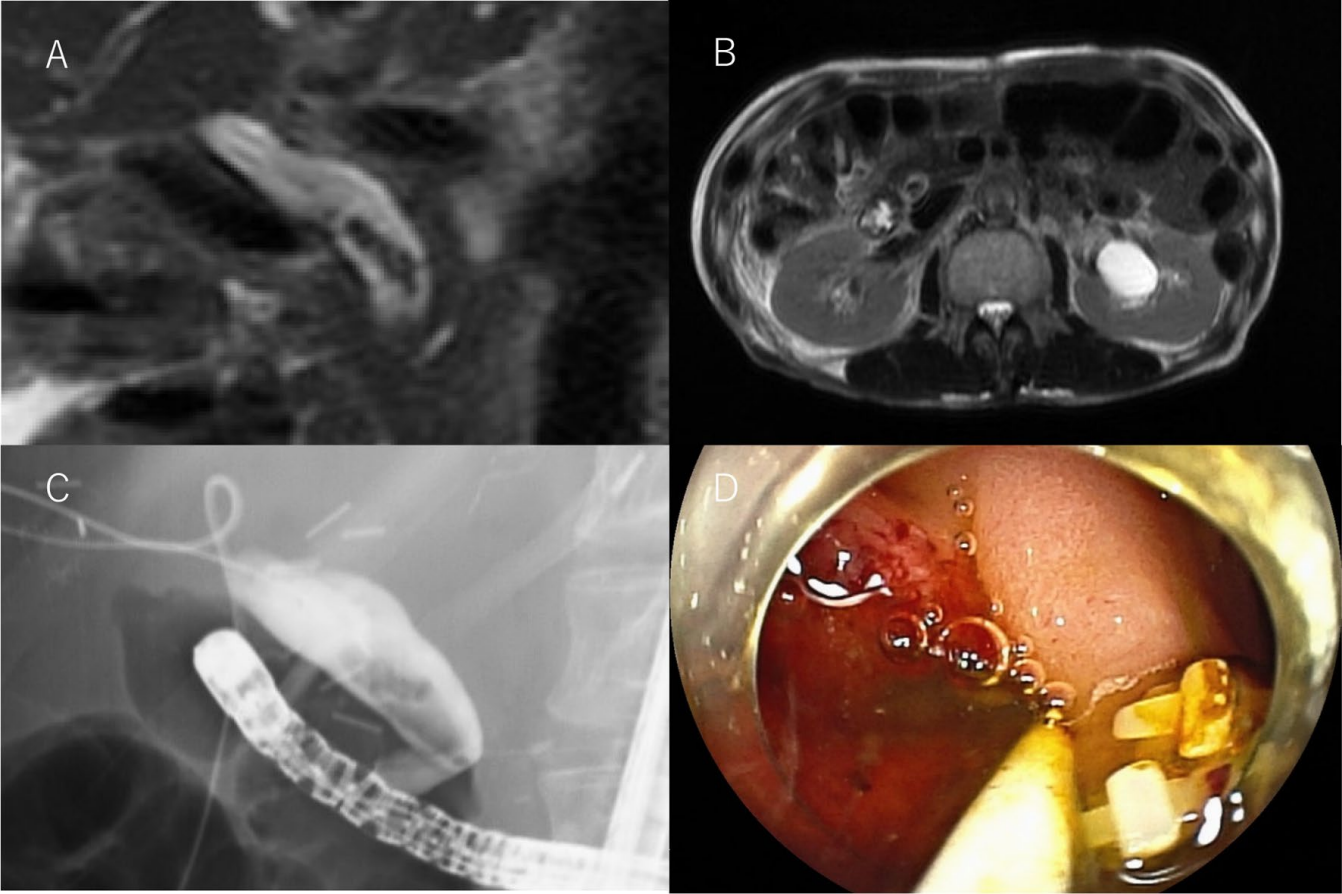
Postoperative findings for case 4. **A**, **B** Postoperative follow-up MRCP image shows common bile duct stones. **C** Endoscopic retrograde cholangiopancreatography image shows common bile duct stones. **D** Two medium-large NAPCs were found with the common bile duct stones

A 74-year-old man had undergone a laparoscopic S5 segmentectomy, an S7 partial liver resection, and a cholecystectomy for liver metastasis from lung cancer and cholelithiasis, at the age of 73 years. A trans-cystic drainage tube was inserted, and it was ligated and fixed in place with NAPCs (Fig. 6). Three months after the operation, a postoperative follow-up MRCP showed common bile duct stones (CBDs; Fig. 7A, B). An ERCP was performed (Fig. 7C), and two NAPCs were found with the CBDs (Fig. 7D). We concluded that the NAPCs that had been placed at the cystic duct had migrated into the common bile duct.

## Discussion

Although previous reports have described metal clip migration after hepato-biliary-pancreatic surgery, very few reports have described a Hem-o-Lok clip migration. We performed a PubMed search with the keywords "Hem-o-Lok” and “migration". The search identified 39 reports. Of these, most involved urology; only 9 involved abdominal surgery. The literature review is summarized in Table 2.

**Table 2:** Summary of cases of NAPC migration after hepato-biliary-pancreatic surgery

Author	Number of patients	Age	Gender	Diagnosis	Operation	Duration of Migration after Initial Surgery	Migrated site	Treatment for the migrated clips	Published	Country
Liu Y. et al. [12]	8	56.3	6 males / 2 females	Cholecystolithiasis and choledocholithiasis	Laparoscopic cholecystectomy and common bile duct exploration	2–3 months	Comon bile duct	Fiberoptic choledochoscope and removed with stone-retrieving basket	2012	China
Seyyedmajidi M. et al. [13]	1	41	Female	Cholecystolithiasis	Laparoscopic cholecystectomy	8 months	Duodenum	Gastrointestinal endoscope and removed using a grasping forceps	2013	Iran
Qu JW. et al. [8]	1	54	Female	Cholecystolithiasis and choledocholithiasis	Laparoscopic cholecystectomy and common bile duct exploration	12 months	Comon bile duct	ERCP, endoscopic sphincterotomy and removed with an extraction balloon	2017	China
Park DJ. et al. [14]	1	58	Male	Early gastric cancer	Laparoscopy-assisted distal gastrectomy with Billroth I gastroduodenostomy	6 months	Anastomosis site	Disappeared Spontaneously	2018	Korea
Kordzadeh A. et al. [15]	1	69	Female	Squamous cell carcinoma of the distal oesophagus	Minimally invasive oesophagectomy with intrathoracic hand-sewn oesophago-gastric anastomosis	3 months	Gastric conduit	Gastroscopy and removed	2018	United Kingdom
Rou WS. et al. [16]	1	53	Female	Cholecystolithiasis	Laparoscopic cholecystectomy	10 months	Comon bile duct	ERCP, endoscopic sphincterotomy and removed with a stone-retrieving basket	2018	Korea
Barabino M. et al. [17]	1	65	Male	Cholecystolithiasis	Laparoscopic cholecystectomy	3 months	Comon bile duct	Roux-en-Y choledochojejunostomy	2018	Italy
Pang L. et al. [18]	6	31	Female	Cholecystolithiasis and choledocholithiasis	Laparoscopic cholecystectomy and common bile duct exploration	4 months	Comon bile duct	Fiberoptic choledochoscope and removed with a stone-retrieving basket	2019	China
		60	Female	Cholecystolithiasis and choledocholithiasis	Laparoscopic cholecystectomy and common bile duct exploration	3 months	T-tube sinus	Fiberoptic choledochoscope and removed with a stone-retrieving basket		
		83	Female	Cholecystolithiasis and choledocholithiasis	Laparoscopic cholecystectomy and common bile duct exploration	6 months	T-tube sinus	Fiberoptic choledochoscope and removed with a stone-retrieving basket		
		61	Female	Chronic cholecystitis	Laparoscopic cholecystectomy		Comon bile duct	Roux-en-Y choledochojejunostomy		
		72	Female	Cholecystolithiasis and choledocholithiasis	Laparoscopic cholecystectomy and common bile duct exploration	4 months	Comon bile duct	Fiberoptic choledochoscope and removed with a stone-retrieving basket		
						18 months	Comon bile duct	Bile duct exploration and J tube drainage		
		64	Female	Cholecystolithiasis and choledocholithiasis	Laparoscopic cholecystectomy and common bile duct exploration	2 months	Comon bile duct	Percutaneous transhepatic biliary drainage and stent implantation		
Roh YJ. et al. [19]	1	65	Male	Cholecystolithiasis	Laparoscopic cholecystectomy	13 months	Comon bile duct	ERCP, endoscopic sphincterotomy and removed with an extraction balloon	2019	Korea

Despite the rarity of this condition, we experienced 4 cases of NAPC migration. Of these 4 cases, 3 occurred after a laparoscopic hepatectomy at our hospital, and 1 occurred after a laparoscopic cholecystectomy at another hospital. In our hospital, NAPCs are generally used for clipping tissues and vessels in a laparoscopic hepatectomy. We performed over 700 laparoscopic hepatectomies between 2010 and 2021. Thus, the 5-year cumulative incidence rate of NAPC migration was 0.5%.

In case 2, the pathological diagnosis was IPT, caused by a migrated NAPC. The concept of IPT was first described in 1954 [1]. Infection, trauma, and foreign bodies are thought to trigger an IPT, but the mechanism remains unclear. In the broad sense, IPTs can arise in multiple disease scenarios, ranging from reactive lesions to true tumorigenesis. In the narrow sense, IPTs arise from repair mechanisms, following an infection or inflammation [2–4]. Pathologically, the IPT is characterized by excessive proliferation of myofibroblasts and fibroblasts with chronic, prominent inflammatory cell infiltration, which mainly comprises lymphocytes and plasma cells [4]. In our case, the lesion was diagnosed as an IPT caused by an inflammatory reaction to a foreign body (the NAPC), followed by host repair mechanisms.

Previous reports have proposed various hypothetical mechanisms for metal clip migration. First, all clips placed near the Calot triangle (e.g., to ligate the cystic duct or to achieve hemostasis of the cystic artery) can potentially migrate into the common bile duct [5]. Second, incomplete closure of the bile duct can lead to the formation of a biloma, where the clip is carried in the bile flow. Then, as the biloma is reabsorbed into the bile duct, the clip is left behind [6]. Third, when a clipped bile duct is compressed by surrounding organs, such as the liver, the clip can migrate into the common bile duct [7]. In lieu of mechanistic evidence, it can be assumed that these mechanisms might also cause NAPC migration. In our cases 2, 3, and 4, NAPC migration occurred after a laparoscopic cholecystectomy; consequently, the mechanisms mentioned above seem likely. For example, in case 2, the operation was performed for acute cholecystitis with choledocholithiasis; thus, the potential mechanism corresponds to the first hypothesis. In case 3, bile was exuded around the common bile duct, due to a lymph node dissection; thus, the potential mechanism corresponds to the second hypothesis. In case 4, a trans-cystic drainage tube was inserted; thus, the potential mechanism also corresponds to the second hypothesis. However, in case 1, NAPC migration occurred after a hepatectomy. This type of migration has not been described previously. Therefore, we hypothesized that NAPC migration occurred due to duodenal adhesion to the resection surface after the hepatectomy.

Previous reports have also described risk factors for clip migration after a laparoscopic cholecystectomy. The risk factors for clip migration included: intraoperative and postoperative complications, cholecystitis with a high degree of inflammation, cholangitis complications, and the use of large numbers of clips [8].

To prevent clip migration, all the technical factors of the surgery should be considered. Accordingly, the relationships of the ducts and vessels in Calot’s triangle should be confirmed during dissection; the number of clips should be minimized, and unnecessary surgical procedures should be avoided [9]. It has also been suggested that clip migration can be avoided using alternative materials or techniques, such as absorbable clips and sutures [6]. In addition, an energy device can be used as an alternative to clips in a laparoscopic cholecystectomy [10, 11]. NAPC migrations related to the laparoscopic cholecystectomy technique can be prevented using laparoscopic coagulation shears (LCS), reducing the number of clips used, and avoiding incomplete closure. On the other hand, in hepatic resections, a large number of clips must be used, due to the large resection surface. Moreover, clips are preferable to LCS for complete closure of the Glissonean pedicle; thus, it is difficult to reduce the number of clips. In that case, the risk of clip migration can be reduced by ensuring that the clips are completely closed and that no clips are dropped.

Based on these findings. we have attempted to avoid clip migration using an LCS or a bipolar coagulation device to seal the blood vessels. In addition, in young patients with benign diseases that have a long postoperative course, we perform ligation, instead of clipping, as much as possible.

## Conclusion

This study contributed to the small amount of evidence currently available on migrated NAPCs. Future investigations are needed to understand the mechanisms of migration. A differential diagnosis of potential complications associated with NAPCs should be considered for patients that have undergone laparoscopic surgery.

## Data Availability

Not applicable.

## Abbreviations

NAPCs: Hem-o-Lok: Non-absorbable polymer clip
CT: Computed tomography
MRCP: Magnetic resonance cholangiopancreatography
ERCP: Endoscopic retrograde cholangiopancreatography
CBDS: Common bile duct stone
IPT: Inflammatory pseudotumor
LCS: Laparoscopic coagulation shears
